# Supplementary material for: Integrating genomic structural equation modeling and experimental validation to unravel the genetic basis of male genital lichen sclerosus
Source: Front Immunol. 2026 Jul 16;17:1857945. doi: 10.3389/fimmu.2026.1857945 (PMC13435250; doi:10.3389/fimmu.2026.1857945)
Supplement: Supplementary file 6 [file SupplementaryFile5.zip › result/fuma_PSYCH_factor_sCCA1_focus.chr9.chr9.14836363.16659655.pop1.pdf]

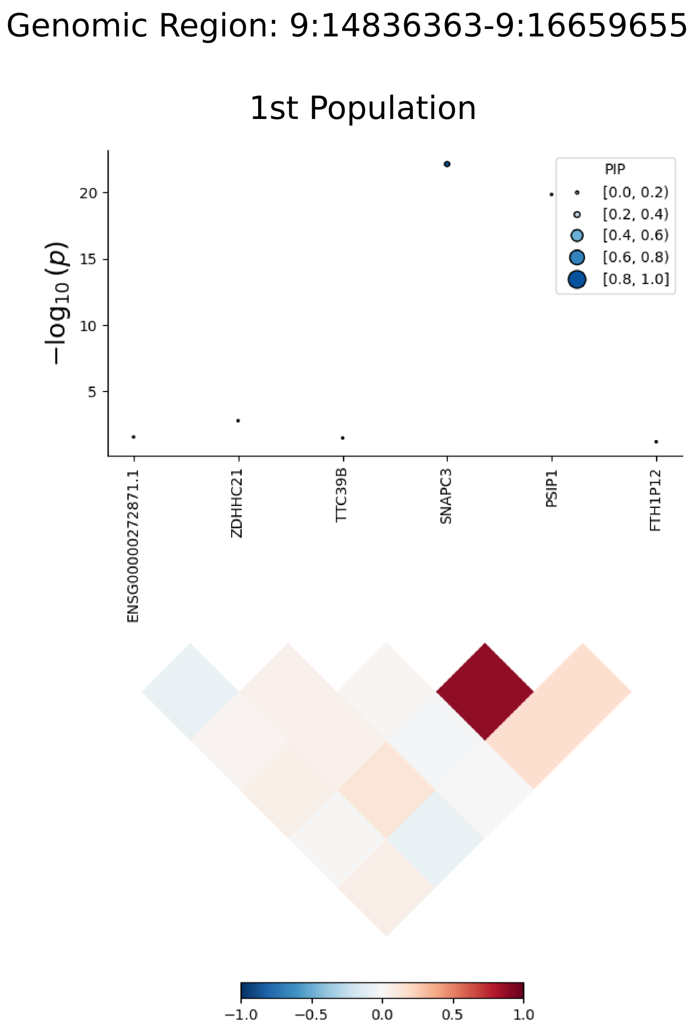

Genomic Region: 9:14836363-9:16659655
1st Population
$-\log_{10}(p)$
20
15
10
5
PIP
[0.0, 0.2)
[0.2, 0.4)
[0.4, 0.6)
[0.6, 0.8)
[0.8, 1.0]
ENSG00000272871.1
ZDHHC21
TTC39B
SNAPC3
PSIP1
FTH1P12
−1.0
−0.5
0.0
0.5
1.0
